# Investigating the health disparities in the association between lifestyle behaviors and the risk of head and neck cancer

**DOI:** 10.1111/cas.14530

**Published:** 2020-07-23

**Authors:** Jenn‐Ren Hsiao, Cheng‐Chih Huang, Chun‐Yen Ou, Chan‐Chi Chang, Wei‐Ting Lee, Sen‐Tien Tsai, Jehn‐Shyun Huang, Ken‐Chung Chen, Yu‐Hsuan Lai, Yuan‐Hua Wu, Wei‐Ting Hsueh, Shang‐Yin Wu, Chia‐Jui Yen, Jang‐Yang Chang, Chen‐Lin Lin, Ya‐Ling Weng, Han‐Chien Yang, Yu‐Shan Chen, Jeffrey S. Chang

**Affiliations:** ^1^ Department of Otolaryngology National Cheng Kung University Hospital College of Medicine National Cheng Kung University Tainan Taiwan; ^2^ Institute of Clinical Medicine College of Medicine National Cheng Kung University Tainan Taiwan; ^3^ Department of Stomatology National Cheng Kung University Hospital College of Medicine National Cheng Kung University Tainan Taiwan; ^4^ Department of Radiation Oncology National Cheng Kung University Hospital College of Medicine National Cheng Kung University Tainan Taiwan; ^5^ Division of Hematology/Oncology Department of Internal Medicine National Cheng Kung University Hospital College of Medicine National Cheng Kung University Tainan Taiwan; ^6^ Department of Nursing National Cheng Kung University Hospital College of Medicine National Cheng Kung University Tainan Taiwan; ^7^ National Institute of Cancer Research National Health Research Institutes Tainan Taiwan

**Keywords:** alcohol, case‐control, head and neck cancer, risk, socioeconomic status

## Abstract

Many studies have reported a positive association between lower socioeconomic status (SES) and higher head and neck cancer (HNC) risk. Fewer studies have examined the impact of SES on the association between alcohol or cigarette use and HNC risk. The current case‐control study (1104 HNC cases and 1363 controls) investigated the influence of education, a SES indicator, on the association between HNC and the use of alcohol, cigarettes, or betel quids in Taiwan, a country with universal health care. Our results showed a larger increase in HNC risk associated with alcohol among those with lower educational level (odds ratio [OR] = 2.07; 95% confidence interval [CI], 1.53‐2.80) than those with higher educational level (OR = 1.38; 95% CI, 1.04‐1.85) (heterogeneity‐*P* = .03). Educational level had an influence on the association between alcohol use and HNC risk among those with genetic susceptibility (*ALDH2*‐deficient) to the carcinogenic effect of alcohol. The association between cigarette or betel quid use and HNC risk was similar between the high and low educational groups. National policies and social interventions have led to the decline in the prevalence of cigarette and betel quid users in Taiwan. In contrast, due to the lack of adequate alcohol control policies, alcohol consumption in Taiwan has continued to rise. A higher impact of alcohol on HNC risk among lower SES individuals even with universal health care could be the result of insufficient alcohol control policies in Taiwan.

## 1 INTRODUCTION

Each year, approximately 710 000 new cases of head and neck cancer (HNC) (cancers of oral cavity, oropharynx, hypopharynx, and larynx) are diagnosed worldwide, making it the seventh most common cancer in the world.^1^ The majority of HNC cases can be attributed to the use of alcohol, betel quids, and cigarettes.^2^ In addition, human papillomavirus has been implicated in the rising incidence of oropharyngeal cancer.^3^ Good oral hygiene habits and diet rich in vegetables and fruits have been associated with a reduced HNC risk.^4^, ^5^

The majority of the risk factors of HNC are modifiable lifestyle factors, which are often influenced by an individual’s socioeconomic status (SES), including educational and income levels. A pooled analysis of 31 case‐control studies, mostly from countries in North America and Europe, showed that two‐thirds of the HNC risk associated with low education could be attributed to the known lifestyle risk factors of HNC; however, a significant inverse association between education and HNC risk still persisted after adjusting for these lifestyle risk factors.^6^ The impact of SES on cancer risk has also been observed by several recent Asian studies. A nationwide multicentered case‐control study with 214 123 Japanese men with cancer and 1 026 247 Japanese male inpatient controls reported that managers in the white‐collar industry tended to have a lower cancer risk compared to the blue‐collar workers, particularly for stomach and lung cancer even after adjusting for the effect of alcohol drinking and smoking.^7^ Another nationwide hospital‐based case‐control study from Japan with 143 806 women with cancer and 703 157 female controls reported that women with higher SES (measured by occupational class) had a reduced risk of cancer overall and the risk of lung and stomach cancer was lower for managers than that of blue‐collar workers in blue‐collar industries, whereas the risk of breast cancer was increased with higher SES, particularly for professionals in the service industry.^8^ A nationwide cohort study following 8 744 603 Korean workers reported an increased risk of esophageal, liver, laryngeal, and lung cancer for men with jobs in service/sales and blue‐collar occupations, while women with jobs in service/sales had an increased risk of cervical cancer. Occupations indicating lower SES were associated with a higher risk of prostate cancer in men, breast, uterine, and ovarian cancer in women, and colorectal, kidney, and thyroid cancer in both men and women.^9^

Although most studies examined SES as an independent risk factor of HNC while adjusting for the lifestyle risk factors of HNC, it is also possible that SES could modify the risk of HNC associated with lifestyle factors. A study from the United States showed that the positive association between alcohol or cigarette use and HNC was stronger for the lower SES group. Studies have also found that SES might influence the relationship between lifestyle factors and cancers other than HNC. For example, a study from Korea showed that heavy alcohol consumption interacted synergistically with low SES to increase the risk of liver cancer.^10^

The current study aimed to investigate the impact of SES on the relationship between the use of alcohol, betel quids, or cigarettes, and HNC risk, using data collected from a case‐control study undertaken in Taiwan. Taiwan is a country with a national health insurance system that began in 1995 and covers 99% of the population. Under this system, citizens of Taiwan have access to low‐cost quality health care with short waiting time.^11^ A study showed that the implementation of the national health insurance narrowed the gap in health disparity in Taiwan.^12^ The current study aimed to evaluate whether, under the national health insurance system, SES could still determine the association between the use of alcohol, betel quids, or cigarettes, and HNC risk.

## 2 MATERIALS AND METHODS

The institutional review boards of the National Health Research Institutes and the National Cheng Kung University Hospital approved the current study. Signed informed consent was obtained from all study participants.

### 2.1 Study subject recruitment

**Figure 1:**
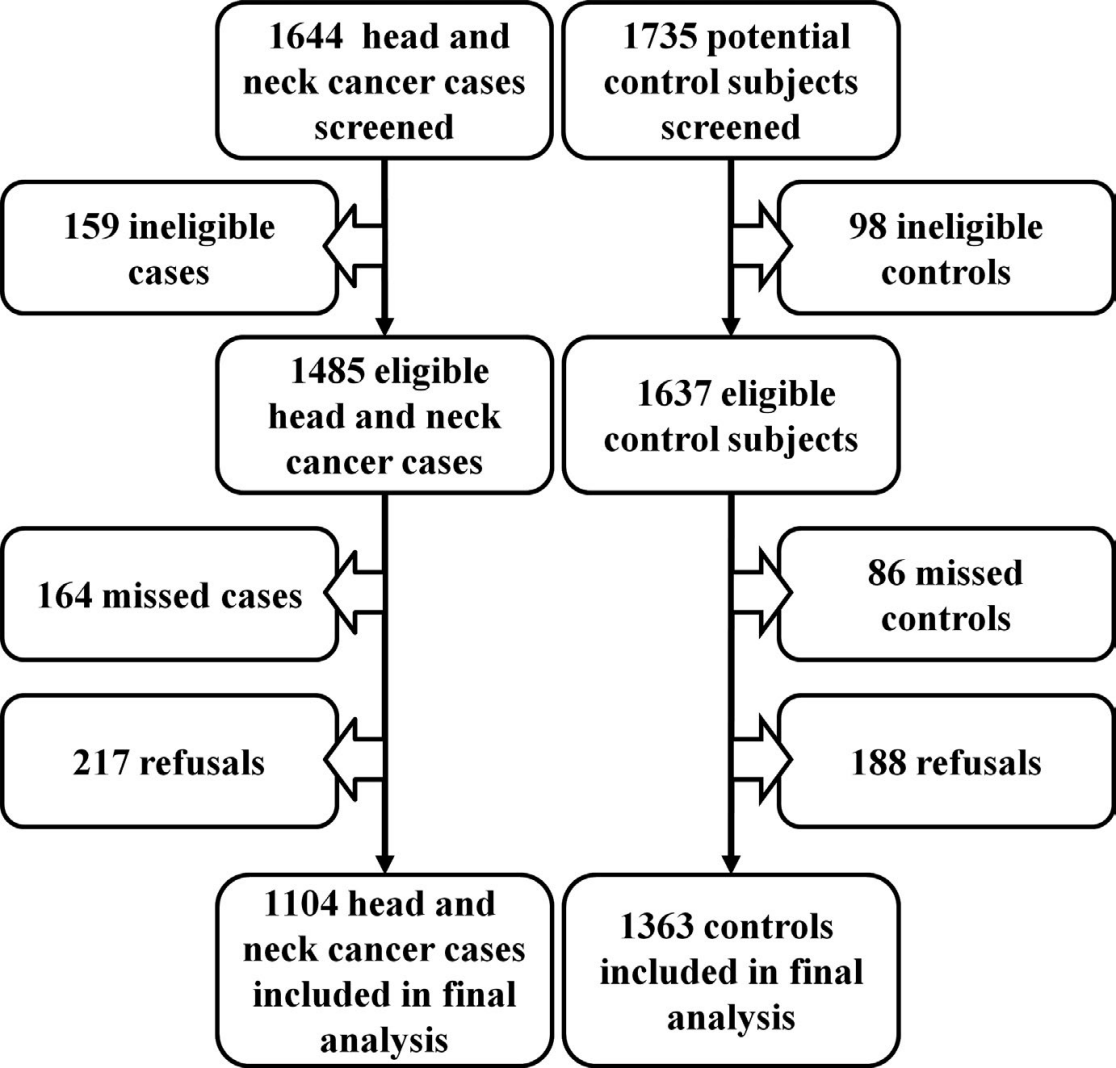
Flowchart for subject recruitment to investigate the health disparities in the association between lifestyle behaviors and the risk of head and neck cancer

This analysis included data collected by an ongoing HNC case‐control study that started recruiting study participants on 1 September 2010 in the Department of Otolaryngology and the Department of Stomatology at the National Cheng Kung University Hospital. Case subjects were individuals with pathologically confirmed squamous cell carcinoma of the head and neck (oral cavity, oropharynx, hypopharynx, and larynx). Control subjects frequency matched to the cases by sex and age were recruited during the same recruitment period as the cases. The recruitment of the case and controls were based on the distribution of 12 sex × age (divided into six 10‐year strata from age 20 to 80 years) groups. The frequency‐matching process was implemented to generate similar distributions of sex and age between the cases and controls. Control subjects were individuals who needed surgery for noncancerous conditions not associated with the use of alcohol, betel quids, or cigarettes. Additional eligibility criteria for both the cases and controls were: (i) no previous diagnosis of cancer; (ii) aged from 20 and 80 years; and (iii) the ability to provide informed consent. The current analysis included subjects recruited during 1 September 2010 to 26 March 2019. Figure 1 presents a flowchart for the subject recruitment. A total of 1644 HNC patients and 1735 potential controls were screened during the recruitment period for the current analysis. The eligible subjects included 1485 HNC cases and 1637 controls. The final samples included 1104 HNC cases (164 missed cases and 217 refusals, participating percentage = 74.3%) and 1363 controls (86 missed controls and 188 refusals, participating percentage = 83.3%). The distribution of the clinical diagnoses of the controls is shown in Table S1.

### 2.2 Interview data collection

Each subject was interviewed to collect information on sex, age, education, income, use of alcohol, betel quids, and cigarettes, oral hygiene habits, and intake of vegetables and fruits. For alcohol drinking, every study participant was initially asked whether she/he ever drank alcoholic beverages. Individuals with a positive response were further asked the following: (i) starting age; (ii) quitting age if the subject had quit drinking alcohol for 6 consecutive months or more; (iii) types of alcoholic beverage (beer, wine, and liquor) consumed; and (iv) drinking frequency (monthly, weekly, or daily) and the number of cups (1 cup = 150 mL, which was the size of the paper cup the interviewer used to show study participants as a reference) drunk each time. For betel quid chewing, each study participant was first asked whether she/he had chewed betel quids at least once per day for 6 consecutive months. Individuals with a positive response were further asked the following: (i) starting age; (ii) quitting age if the subject had quit chewing betel quids for 6 consecutive months or more; and (iii) the number of betel quids chewed per day. For cigarette smoking, each study participant was first asked whether she/he had smoked at least 100 cigarettes during her/his lifetime. Individuals with a positive response were further asked the following: (i) starting age; (ii) quitting age if the subject had quit smoking cigarettes for 6 consecutive months or more; and (iii) the number of cigarettes smoked per day. For oral hygiene, each participant was asked about: (i) regular dental visits (yes/no and frequency); (ii) tooth brushing (number of times per day); and (iii) use of dental floss (yes/no). For the intake of vegetables and fruits, each subject was asked about the frequency of fresh vegetable or fruit intake (once/week or less, 2‐4 times/week, or daily). Good oral hygiene and higher intake of vegetables and fruits have been associated with a reduced HNC risk.^4^, ^5^ Furthermore, oral hygiene and intake of fresh of vegetables and fruits could be associated with SES according to our data (Table S2). Therefore, oral hygiene and intake of fresh vegetables and fruits were included in the current analysis for 2 purposes: (i) when considering the association between education or income (markers of SES) and HNC risk, oral hygiene and intake of vegetables and fruits along with use of alcohol, betel quids, and cigarettes were considered as mediators because SES might determine these lifestyle behaviors and adjusting for these mediators in the model allowed us to see whether there could be other unmeasured SES‐related risk factors contributing to the association between SES and HNC risk; and (ii) when examining the association between the use of alcohol, betel quids, and cigarettes and HNC by educational level, oral hygiene and intake of vegetables and fruits were treated as potential confounders.

### 2.3 Blood sample collection

Pretreatment blood samples were collected from study subjects using EDTA‐containing vacutainer tubes. Centrifugation of the blood samples was carried out to separate out the buffy coat. DNA extraction from the buffy coat was undertaken using a commercially available DNA purification kit. DNA samples were kept in a refrigerator at −80°C until ready to use.

### 2.4 Genotyping of *ALDH2* rs671

Because the metabolism of acetaldehyde, a well‐established carcinogen generated through alcohol metabolism, is determined by the genotype of the *ALDH2* gene, we decided to investigate whether the health inequality in the association between alcohol and HNC risk is more prominent among genetically susceptible individuals. *ALDH2* has a well‐known functional single nucleotide polymorphism (SNP), rs671.^13^ The ALDH2*1/*2 genotype and the ALDH2*2/*2 genotype encode an enzyme with less than 50% and 4% enzyme activity, respectively, compared to the enzyme encoded by the ALDH2*1/*1 WT genotype.^14^ *ALDH2* rs671 was genotyped for each study participant using the TaqMan‐based allelic discrimination method on a 7500 Real‐Time Polymerase Chain Reaction System (Applied Biosystems). To minimize genotyping error, 10% of the samples were randomly chosen for duplicate genotyping and the results showed 100% concordance.

### 2.5 Statistical analysis

The χ^2^ tests (for categorical variables) and *t* tests (for continuous variables) were used to compare the distributions of sex, age, education, income, use of alcohol, betel quids, and cigarettes, oral hygiene, and intake of fresh vegetables and fruits.

Because the study is still ongoing with continued recruitment of study subjects, this might have resulted in some small imbalances between cases and controls in the distributions of age and sex at the time of data analysis for the current study. In addition, because age was frequency matched in a 10‐year range, the average age for the cases and the controls might not be exactly the same. The differences in the distribution of age and sex were adjusted by including the age and the sex variables in the multivariate logistic regression models. The association between educational levels and HNC or between income levels and HNC was analyzed separately. Unconditional logistic regression analysis was carried out to calculate the odds ratio (OR) and 95% confidence interval (CI) for the association between education or income levels and HNC risk, adjusted for age and sex in Model 1 and adjusted for age, sex, use of alcohol, betel quids, and cigarettes, oral hygiene, and intake of vegetables and fruits in Model 2. As mentioned above, lifestyle factors, including use of alcohol, betel quids, and cigarettes, oral hygiene, and intake of vegetables and fruits could act as mediators on the pathway for the association between SES and HNC. The purpose for adjusting for these lifestyle factors in Model 2 was to evaluate for the possible existence of additional unmeasured lifestyle factors mediating the relationship between SES and HNC.

Because approximately 30% of the study subjects refused to answer the question on income, further analyses for the influence of SES on the association between the use of alcohol, betel quids, or cigarettes and HNC risk were only carried out with the education variable.

To evaluate the impact of education on the association between the use of alcohol, betel quids, or cigarettes and HNC risk, the relationship between the use of alcohol, betel quids, or cigarettes and HNC risk was analyzed stratified by education level (junior high school or lower vs high school/technical school or higher). We dichotomized educational level to minimize the reduction in statistical power in the stratified analysis. Furthermore, we divided the educational level into junior high or lower vs high school/technical school or higher because our analysis indicated that those with an education level at junior high or lower had an increased HNC risk compared to those with college or higher education, whereas the HNC risk was similar between those with high school/technical school education and those with college or higher education. Heterogeneity between the 2 education strata was assessed by comparing the full statistical model with the product term (education × alcohol or education × betel quid or education × cigarette) to the model without the product term using the log‐likelihood ratio test. Level‐specific heterogeneity test was carried out to assess the difference by education for each level of alcohol, betel quid, or cigarette use. In addition, overall heterogeneity test was used to evaluate the combined difference by education across the different levels of alcohol, betel quid, or cigarette use. Alcohol use was evaluated by: (i) the status of drinking: never + occasional drinker, former regular drinker, and current regular drinker with regular drinking defined as drinking alcohol at least once per week; (ii) the frequency of drinking: never, monthly, weekly, and daily; and (iii) the level of drinking: none = 0 g/d, light drinking is less than 14 g/d (less than 1 drink per day), moderate drinking = 14‐42 g/d (1‐3 drinks/d), and heavy drinking is >42 g/d (more than 3 drinks/d). Fourteen grams of alcohol = 1 drink according to the National Institute on Alcohol Abuse and Alcoholism (https://www.niaaa.nih.gov/what‐standard‐drink). The definition for the level of drinking was based on those commonly adopted by other studies.^15^, ^16^ Data on the types of alcoholic beverage consumed and the volume and the frequency of drinking were used to calculate the grams of alcohol per day using the formula: total volume of alcohol per day × alcohol content × 0.798 g/mL (this is the density of ethanol). The alcohol content was set at 5%, 13%, and 40% for beer, wine, and liquor, respectively. The total grams of alcohol per day were calculated by summing up the grams per day of alcohol from the different types of alcoholic beverage. Betel quid use was examined by: (i) the status of chewing: never, former, or current; and (ii) the pack‐years of chewing with 1 pack‐year = 1 pack of betel quids (20 quids) use per day × 1 year. Cigarette use was examined by: (i) the status of smoking: never, former, or current; and (ii) the pack‐years of smoking with 1 pack‐year = 1 pack of cigarettes (20 cigarettes) use per day × 1 year.

To evaluate whether genetic background might affect the influence of educational level on the association between alcohol use and HNC risk, the analysis on the association between alcohol and HNC was further stratified by *ALDH2* genotype (ALDH2*1/*1 [normal function] vs ALDH2*1/*2 or ALDH2*2/*2 [slow or nonfunctional]) in addition to the stratification by educational level.

## 3 RESULTS

The current analysis included 1104 HNC patients and 1363 controls (Table 1). The analysis with the level of alcohol use (never, light, moderate, and heavy) included fewer subjects (1030 cases and 1295 controls), because the data on alcoholic beverage type were not collected until after 20 March 2011. Cases were slightly older than controls (mean age, 55.9 years vs 54.7 years, *P* = .005). Ninety‐four percent of the study subjects were men, although the HNC group had a higher proportion of women than the control group (7.1% vs 4.5%, *P* = .006). Controls had higher levels of education and income compared to the cases. A higher proportion of cases were users of alcohol, betel quids, or cigarettes compared to the controls. Controls had better oral hygiene habits and ate vegetables and fruits more frequently than the cases.

**Table 1:** Demographic and lifestyle characteristics of head and neck cancer patients and control subjects

Characteristics	Cases, N = 1104 n (%)	Controls, N = 1363 n (%)	*P* value^a^
Age (years)			
Mean (SE)	55.9 (0.3)	54.7 (0.3)	.0050
Sex			
Men	1026 (92.9)	1302 (95.5)	.0060
Women	78 (7.1)	61 (4.5)	
Education			
≤Elementary school	296 (26.8)	215 (15.8)	<.0001
Junior high	323 (29.3)	232 (17.0)	
High school/technical school	366 (33.1)	482 (35.4)	
College or higher	119 (10.8)	434 (31.8)	
Monthly income			
NT$ <20 000	172 (15.6)	123 (9.0)	<.0001
NT$ 20 000‐39 999	192 (17.4)	135 (9.9)	
NT$ 40 000‐59 999	165 (14.9)	188 (13.8)	
NT$ 60 000‐79 999	93 (8.4)	159 (11.7)	
NT$ 80 000‐99 999	44 (4.0)	87 (6.4)	
NT$ > 100 000	85 (7.7)	305 (22.4)	
Unknown	353 (32.0)	366 (26.8)	
Alcohol drinking			
Never + occasional	367 (33.2)	785 (57.6)	<.0001
Former regular	165 (15.0)	133 (9.8)	
Current regular	571 (51.7)	445 (32.6)	
Unknown	1 (0.1)	0 (0.0)	
Never	330 (29.9)	708 (51.9)	<.0001
Monthly	37 (3.3)	77 (5.7)	
Weekly	118 (10.7)	184 (13.5)	
Daily	582 (52.7)	381 (27.9)	
Unknown	37 (3.3)	13 (1.0)	
Never^b^	314 (30.5)	689 (53.2)	<.0001
Light	161 (15.6)	271 (20.9)	
Moderate	148 (14.4)	151 (11.7)	
Heavy	369 (35.8)	167 (12.9)	
Unknown	38 (3.7)	17 (1.3)	
Mean grams/day (SE)	51.5 (2.8)	19.8 (1.5)	<.0001
Betel quid chewing			
Never	311 (28.2)	985 (72.3)	<.0001
Former	431 (39.0)	251 (18.4)	
Current	362 (32.8)	125 (9.2)	
Unknown	0 (0.0)	2 (0.1)	
0 pack‐years	311 (28.2)	985 (72.3)	<.0001
0.1‐17 pack‐years	262 (23.7)	187 (13.7)	
>17 pack‐years	508 (46.0)	186 (13.7)	
Unknown	23 (2.1)	5 (0.3)	
Mean pack‐years (SE)	28.9 (1.6)	7.0 (0.5)	<.0001
Cigarette smoking			
Never	162 (14.7)	470 (34.5)	<.0001
Former	211 (19.1)	289 (21.2)	
Current	730 (66.1)	603 (44.2)	
Unknown	1 (0.1)	1 (0.1)	
0 pack‐years	162 (14.7)	470 (34.5)	<.0001
0.1‐29.3 pack‐years	324 (29.3)	442 (32.4)	
>29.3 pack‐years	606 (54.9)	442 (32.4)	
Unknown	12 (1.1)	9 (0.7)	
Mean pack‐years (SE)	35.9 (0.9)	22.2 (0.7)	<.0001
Oral hygiene score^c^			
0, 1 (Good)	247 (22.4)	628 (46.1)	<.0001
2 (Moderate)	437 (39.6)	503 (36.9)	
3 (Poor)	415 (37.6)	231 (16.9)	
Unknown	5 (0.4)	1 (0.1)	
Fresh vegetables			
≤Once/week	39 (3.5)	17 (1.2)	<.0001
2‐4 times/week	145 (13.1)	95 (7.0)	
Daily	918 (83.2)	1,251 (91.8)	
Unknown	2 (0.2)	0 (0.0)	
Fresh fruits			
≤Once/week	463 (41.9)	305 (22.4)	<.0001
2‐4 times/week	286 (25.9)	352 (25.8)	
Daily	352 (31.9)	705 (51.7)	
Unknown	3 (0.3)	1 (0.1)	

^a^ Calculated using χ^2^ test excluding the unknowns.

^b^ Light, <14 g/d; moderate, 14‐42 g/d; heavy, >42 g/d.

^c^ Oral hygiene score = tooth brushing + use of dental floss + regular dental visit, with tooth brushing: ≤2 times per day = 0, <2 times per day = 1; Use of dental floss: yes = 0, no = 1; and regular dental visit: yes = 0, no = 1.

Lower educational and income levels were associated with an increased HNC risk even after adjusting for age, sex, use of alcohol, betel quids, and cigarettes, oral hygiene, and consumption of vegetables and fruits (Table 2). Compared to those with an educational level of college or higher, an increased HNC risk was observed among those with an educational level of junior high (OR = 1.52; 95% CI, 1.11‐2.08) or elementary school or lower (OR = 1.42; 95% CI, 1.00‐2.01). An increasing trend in HNC risk was observed with decreasing income levels.

**Table 2:** Association between socioeconomic factors and head and neck cancer (HNC) risk

Characteristics	Cases n (%)	Controls n (%)	Model 1^a^, ^c^ OR (95% CI)	Model 2^b^, ^c^ OR (95% CI)
Education				
College or higher	119 (10.8)	434 (31.8)	Reference	Reference
High school/technical school	366 (33.1)	482 (35.4)	2.78 (2.18‐3.55)	1.16 (0.87‐1.55)
Junior high	323 (29.3)	232 (17.0)	5.16 (3.96‐6.73)	1.52 (1.11‐2.08)
≤Elementary school	296 (26.8)	215 (15.8)	5.33 (3.97‐7.17)	1.42 (1.00‐2.01)
			*P*‐trend < .0001	*P*‐trend = .0100
Income				
NT$ >100 000	85 (7.7)	305 (22.4)	Reference	Reference
NT$ 80 000‐99 999	44 (4.0)	87 (6.4)	1.82 (1.18‐2.82)	1.87 (1.13‐3.08)
NT$ 60 000‐79 999	93 (8.4)	159 (11.7)	2.14 (1.51‐3.04)	1.76 (1.18‐2.63)
NT$ 40 000‐59 999	165 (14.9)	188 (13.8)	3.21 (2.33‐4.42)	2.04 (1.42‐2.93)
NT$ 20 000‐39 999	192 (17.4)	135 (9.9)	5.14 (3.71‐7.13)	2.62 (1.80‐3.81)
NT$ <20 000	172 (15.6)	123 (9.0)	5.05 (3.55‐7.05)	2.20 (1.49‐3.25)
Unknown	353 (32.0)	366 (26.8)	—	—
			*P*‐trend < .0001	*P*‐trend < .0001

—, not applicable.

^a^ Odds ratio (OR) and 95% confidence interval (CI) were calculated using unconditional logistic regression, adjusted for age and sex.

^b^ OR and 95% CI were calculated using unconditional logistic regression, adjusted for age, sex, use of alcohol (frequency), betel quids (pack‐years), cigarette (pack‐years), oral hygiene score, and consumption of vegetables and fruits.

^c^ Association between educational levels and HNC or between income levels and HNC was analyzed separately.

The positive association between alcohol and HNC risk was stronger among the lower educational group compared to the higher educational group (ever vs never/occasional drinking for educational level ≤ junior high school: OR = 2.07; 95% CI, 1.53‐2.80; high school or higher: OR = 1.38; 95% CI, 1.04‐1.85; heterogeneity‐*P* = .03), particularly for current regular drinkers (junior high school or lower: OR = 2.54; 95% CI, 1.83‐3.53; high school or higher: OR = 1.29; 95% CI, 0.95‐1.74; heterogeneity‐*P* = .0001) (Table 3). The risk of HNC increased significantly at the lower frequency and the amount of alcohol use for the lower educational group compared to that of the higher educational group. In terms of frequency of alcohol drinking, a significantly increased HNC risk was observed for weekly (OR = 1.89; 95% CI, 1.17‐3.03) or daily (OR = 2.12; 95% CI, 1.52‐2.96) drinking in the lower educational group but only for daily drinking (OR = 1.51; 95% CI, 1.09‐2.09) in the higher educational group. For the level of alcohol drinking, a significantly increased HNC risk was seen for moderate (OR = 1.83; 95% CI, 1.15‐2.90) and heavy drinking (OR = 2.61; 95% CI, 1.75‐3.87) in the lower educational group. For the higher educational group, an increased HNC risk was observed only for heavy drinking (OR = 2.20; 95% CI, 1.49‐3.25). The association between cigarette or betel quid use and HNC risk was similar between the high and low educational groups with no clear difference according to the different levels of cigarette or betel quid use.

**Table 3:** Association between the use of alcohol, betel quids, and cigarettes and head and neck cancer risk by the level of education

Lifestyle factors	Education level ≤ junior high			Education level ≥ high school/technical school			Level‐specific heterogeneity‐*P*
	Cases, N = 619 n (%)	Controls, N = 447 n (%)	OR (95% CI)^a^	Cases, N = 485 n (%)	Controls, N = 916 n (%)	OR (95% CI)^a^	
Alcohol drinking							
Never + occasional	189 (30.5)	245 (54.8)	Reference	178 (36.7)	540 (58.9)	Reference	
Former regular	98 (15.8)	70 (15.7)	1.29 (0.85‐1.96)	67 (13.8)	63 (6.9)	1.83 (1.16‐2.88)	.2900
Current regular	332 (53.6)	132 (29.5)	2.54 (1.83‐3.53)	239 (49.3)	313 (34.2)	1.29 (0.95‐1.74)	.0001
Unknown	0 (0.0)	0 (0.0)	—	1 (0.2)	0 (0.0)	—	
	Overall heterogeneity‐*P* ^b^ = .0004						
Ever (former + current regular)	430 (69.5)	202 (45.2)	2.07 (1.53‐2.80)	306 (63.2)	376 (41.1)	1.38 (1.04‐1.85)	.0300
	Overall heterogeneity‐*P* ^b^ = .03						
Never	173 (27.9)	222 (49.7)	Reference	157 (32.4)	486 (53.0)	Reference	
Monthly	16 (2.6)	23 (5.1)	1.03 (0.49‐2.18)	21 (4.3)	54 (5.9)	1.50 (0.82‐2.76)	.6100
Weekly	65 (10.5)	49 (11.0)	1.89 (1.17‐3.03)	53 (10.9)	135 (14.7)	1.16 (0.75‐1.77)	.0500
Daily	345 (55.7)	146 (32.6)	2.12 (1.52‐2.96)	237 (48.9)	235 (25.7)	1.51 (1.09‐2.09)	.1100
Unknown	20 (3.2)	7 (1.6)	—	17 (3.5)	6 (0.7)	—	
	Overall heterogeneity‐*P* ^b^ = .13						
Never^c^	163 (28.5)	212 (50.2)	Reference	151 (33.0)	477 (54.6)	Reference	
Light	83 (14.5)	78 (18.5)	1.44 (0.94‐2.21)	78 (17.0)	193 (22.1)	1.16 (0.80‐1.69)	.2900
Moderate	88 (15.4)	50 (11.9)	1.83 (1.15‐2.90)	60 (13.1)	101 (11.6)	1.19 (0.76‐1.86)	.1300
Heavy	217 (37.9)	74 (17.5)	2.61 (1.75‐3.87)	152 (33.2)	93 (10.7)	2.20 (1.49‐3.25)	.4900
Unknown	21 (3.7)	8 (1.9)	—	17 (3.7)	9 (1.0)	—	
	Overall heterogeneity‐*P* ^b^ =.43						
Betel quid chewing							
Never	148 (23.9)	266 (59.5)	Reference	163 (33.6)	719 (78.5)	Reference	
Former	253 (40.9)	119 (26.6)	3.76 (2.65‐5.33)	178 (36.7)	132 (14.4)	4.60 (3.28‐6.45)	.8200
Current	218 (35.2)	61 (13.6)	5.85 (3.84‐8.91)	144 (29.7)	64 (7.0)	7.92 (5.30‐11.83)	.8000
Unknown	0 (0.0)	1 (0.2)	—	0 (0.0)	1 (0.1)	—	
	Overall heterogeneity‐*P* ^b^ = .96						
Ever (former + current)	471 (76.1)	180 (40.2)	4.36 (3.15‐6.04)	322 (66.4)	196 (21.4)	5.59 (4.11‐7.61)	.7600
	Overall heterogeneity‐*P* ^b^ = .76						
0 pack‐years	148 (23.9)	266 (59.5)	Reference	163 (33.6)	719 (78.5)	Reference	
0.1‐17 pack‐years	135 (21.8)	73 (16.3)	3.22 (2.16‐4.79)	127 (26.2)	114 (12.5)	4.35 (3.04‐6.21)	.7700
>17 pack‐years	320 (51.7)	105 (23.5)	5.16 (3.59‐7.41)	188 (38.8)	81 (8.8)	6.99 (4.80‐10.17)	.4600
Unknown	16 (2.6)	3 (0.7)	—	7 (1.4)	2 (0.2)	—	
	Overall heterogeneity‐*P* ^b^ = .77						
Cigarette smoking							
Never	78 (12.6)	127 (28.4)	Reference	84 (17.3)	343 (37.4)	Reference	
Former	125 (20.2)	102 (22.8)	1.50 (0.92‐2.44)	86 (17.7)	187 (20.4)	1.43 (0.92‐2.21)	.4700
Current	415 (67.0)	218 (48.8)	1.33 (0.86‐2.15)	315 (65.0)	385 (42.0)	1.45 (0.97‐2.16)	.5300
Unknown	1 (0.2)	0 (0.0)	—	0 (0.0)	1 (0.1)	—	
	Overall heterogeneity‐*P* ^b^ = .75						
Ever (former + current)	540 (87.2)	320 (71.6)	1.41 (0.92‐2.18)	401 (82.7)	572 (62.4)	1.44 (0.99‐2.10)	.4600
	Overall heterogeneity‐*P* ^b^ = .46						
0 pack‐years	78 (12.6)	127 (28.4)	Reference	84 (17.3)	343 (37.4)	Reference	
0.1‐29.3 pack‐years	159 (25.7)	117 (26.2)	1.60 (0.99‐2.58)	165 (34.0)	325 (35.5)	1.39 (0.93‐2.07)	.1700
>29.3 pack‐years	374 (60.4)	201 (45.0)	1.27 (0.80‐2.02)	232 (47.8)	241 (26.3)	1.53 (1.00‐2.32)	.9100
Unknown	8 (1.3)	2 (0.4)	—	4 (0.8)	7 (0.8)	—	
	Overall heterogeneity‐*P* ^b^ = .23						

—, not applicable.

^a^ Odds ratio (OR) and 95% confidence interval (CI) were calculated using unconditional logistic regression, adjusted for age, sex, oral hygiene score, and consumption of vegetables and fruits, betel quids (number of quids per day) and cigarette (number of cigarettes per day) was made for analysis with alcohol. Additional adjustment for use of alcohol (frequency) and cigarette (pack‐years) was made for analysis with betel quids. Additional adjustment for the use of alcohol (frequency) and betel quids (pack‐years) was made for analysis with cigarettes.

^b^ Overall heterogeneity‐*P* evaluated the combined difference by education across the different levels of the lifestyle factors. The heterogeneity‐*P* was calculated excluding the unknowns.

^c^ Light, <14 g/d; moderate, 14‐42 g/d; heavy, >42 g/d.

For individuals with the *ALDH2*‐normal genotype, alcohol was not significantly associated with HNC risk regardless of the educational level (Table 4). For individuals carrying the *ALDH2*‐deficient genotype, the positive association between alcohol drinking and HNC risk was more prominent in the lower educational group. In the *ALDH2*‐deficient genotype group, an increased HNC risk was observed at the weekly and daily drinking levels for the lower educational group but only at the daily drinking level for the higher educational group. When analyzed by the level of alcohol use, a statistically significant increased HNC risk could be observed already at the light drinking level for the lower educational group, whereas for the higher educational group, heavy drinking was needed to see a significant association with HNC. These results suggested that educational level had an influence on the association between alcohol use and HNC risk among those with genetic predisposition to develop alcohol‐related HNC.

**Table 4:** Association between alcohol use and head and neck cancer risk by level of education and *ALDH2* genotype

	ALDH2*1/*1 (normal function)			ALDH2*1/*2 or *2/*2 (slow or nonfunctional)		
	Education level ≤ junior high	Education level ≥ high school/ technical school	Level‐specific heterogeneity‐*P*	Education level ≤ junior high	Education level ≥ high school/technical school	Level‐specific heterogeneity‐*P*
	OR (95% CI)^a^	OR (95% CI)^a^		OR (95% CI)^a^	OR (95% CI)^a^	
Alcohol drinking						
Never + occasional	Reference	Reference		Reference	Reference	
Former regular	0.68 (0.32‐1.44)	1.57 (0.77‐3.16)	0.06	1.83 (1.02‐3.27)	1.98 (1.04‐3.77)	.97
Current regular	1.21 (0.64‐2.28)	0.74 (0.45‐1.23)	0.23	4.32 (2.74‐6.80)	2.80 (1.81‐4.33)	.14
	Overall heterogeneity‐*P* ^b^ = .01			Overall heterogeneity‐*P* ^b^ = .30		
Ever (former + current)	1.02 (0.55‐1.89)	0.86 (0.53‐1.39)	0.71	3.32 (2.23‐4.96)	2.56 (1.72‐3.83)	.28
	Overall heterogeneity‐*P* ^b^ = .71			Overall heterogeneity‐*P* ^b^ = .28		
Never	Reference	Reference		Reference	Reference	
Monthly	0.99 (0.30‐3.29)	1.41 (0.57‐3.47)	0.57	1.03 (0.37‐2.90)	1.66 (0.70‐3.94)	.71
Weekly	0.97 (0.43‐2.17)	0.81 (0.42‐1.57)	0.75	3.15 (1.51‐6.56)	1.59 (0.84‐3.01)	.08
Daily	1.01 (0.49‐2.09)	0.91 (0.52‐1.58)	0.90	3.42 (2.21‐5.32)	3.22 (2.03‐5.11)	.74
	Overall heterogeneity‐*P* ^b^ = .90			Overall heterogeneity‐*P* ^b^ = .33		
Never^c^	Reference	Reference		Reference	Reference	
Light	0.82 (0.36‐1.84)	0.86 (0.48‐1.54)	0.67	2.03 (1.14‐3.62)	1.71 (0.98‐2.99)	.29
Moderate	0.96 (0.42‐2.18)	0.89 (0.45‐1.76)	0.96	2.86 (1.44‐5.66)	1.61 (0.82‐3.14)	.22
Heavy	1.26 (0.57‐2.79)	1.07 (0.56‐2.04)	0.97	4.22 (2.44‐7.30)	6.63 (3.58‐12.29)	.31
	Overall heterogeneity‐*P* ^b^ = .96			Overall heterogeneity‐*P* ^b^ = .22		

^a^ Odds ratio (OR) and 95% confidence interval (CI) were calculated using unconditional logistic regression, adjusted for age, sex, oral hygiene score, consumption of vegetables and fruits, and use of betel quids (pack‐years) and cigarette (pack‐years).

^b^ Overall heterogeneity‐*P* evaluated the combined difference by education across the different levels of the lifestyle factors. The heterogeneity‐*P* was calculated excluding the unknowns.

^c^ Light, <14 g/d; moderate, 14‐42 g/d; heavy, >42 g/d.

The magnitude of the positive association between alcohol drinking itself or in combination with cigarette smoking and/or betel quid chewing and HNC risk was larger in the lower educational group compared to that in the higher educational group (Table 5).

**Table 5:** Association between the use of alcohol, betel quids, cigarettes in combination and head and neck cancer risk by the level of education

Use of alcohol, betel quids, and cigarettes	Education level ≤ junior high			Education level ≥ high school/technical school		
	Cases, N = 619 n (%)	Controls, N = 447 n (%)	OR (95% CI)^a^	Cases, N = 485 n (%)	Controls, N = 916 n (%)	OR (95% CI)^a^
No use	39 (6.3)	103 (23.0)	Reference	61 (12.6)	274 (29.9)	Reference
Alcohol only	8 (1.3)	17 (3.8)	2.85 (1.01‐8.04)	7 (1.4)	59 (6.4)	0.89 (0.38‐2.14)
Alcohol + betel quids or alcohol + cigarettes	83 (13.4)	77 (17.2)	6.57 (3.32‐12.99)	63 (13.0)	192 (21.0)	2.26 (1.41‐3.62)
Alcohol + betel quids + cigarettes	338 (54.6)	108 (24.2)	18.95 (9.85‐36.45)	236 (48.7)	124 (13.5)	10.75 (6.86‐16.84)
Other combinations	150 (24.2)	141 (31.5)	6.50 (3.41‐12.40)	117 (24.1)	265 (28.9)	2.88 (1.86‐4.45)
Unknown	1 (0.2)	1 (0.2)	—	1 (0.2)	2 (0.2)	—
	Overall heterogeneity‐*P* ^b^ = .02					

—, not applicable.

^a^ Odds ratio (OR) and 95% confidence interval (CI) were calculated using unconditional logistic regression, adjusted for age, sex, oral hygiene score, and consumption of vegetables and fruits

^b^ Overall heterogeneity‐*P* evaluated the combined difference by education across the different levels of the lifestyle factors. The heterogeneity‐*P* was calculated excluding the unknowns.

## 4 DISCUSSION

Our results showed that lower educational and income levels were associated with an increased HNC risk, even after adjusting for the use of alcohol, betel quids, and cigarettes, oral hygiene, and intake of vegetables and fruits. The positive association between alcohol drinking and HNC risk was stronger among the lower educational group. Educational level did not influence the association between the use of betel quids or cigarettes and HNC risk. Educational level had more influence on the association between alcohol and HNC risk among those genetically susceptible to the carcinogenic effect of alcohol.

**Figure 2:**
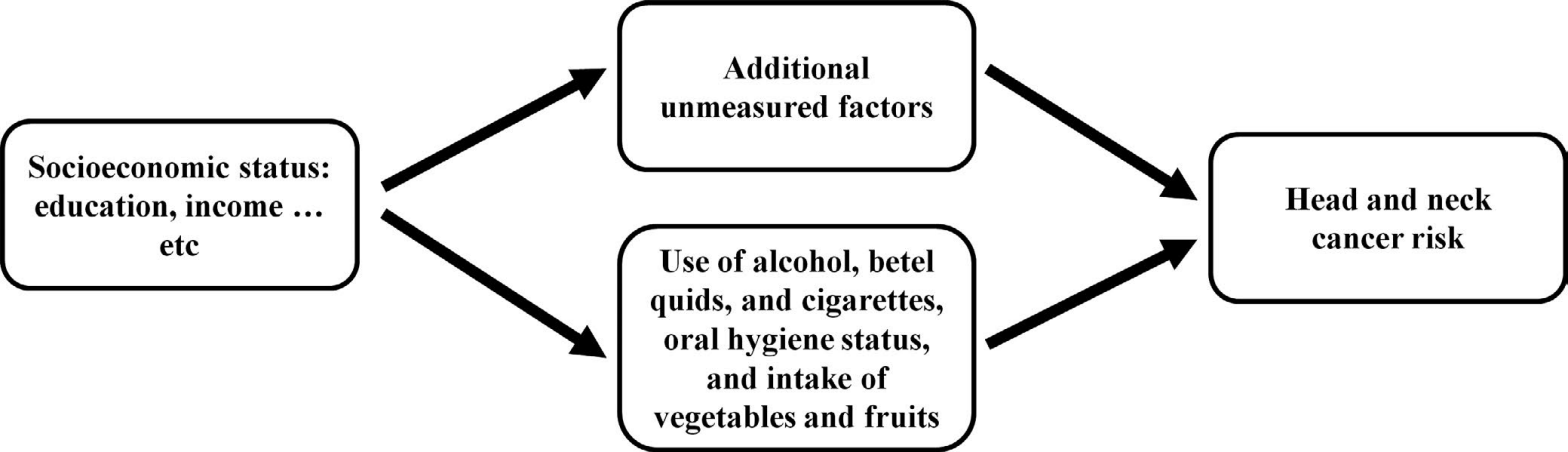
Directed acyclic graph indicating the mediating roles of use of alcohol, betel quids, and cigarettes, oral hygiene status, intake of vegetables and fruits, and additional unmeasured factors on the pathway for the association between socioeconomic status (eg education, income) and head and neck cancer

Consistent with results of previous studies, our analysis showed that lower SES, including lower educational and income levels, was associated with a higher HNC risk. The association remained statistically significant after adjusting for the use of alcohol, betel quids, and cigarettes, oral hygiene, and intake of vegetables and fruits, although the association was substantially attenuated. In a multicenter case‐control study with 2198 upper aerodigestive tract cancer (UADT cancer = HNC + esophageal cancer) cases and 2141 controls, Conway et al showed that, compared to those with university level education, those with less than university education had a significantly elevated risk of UADT cancer.^17^ However, after adjusting for alcohol drinking, cigarette smoking, and consumption of fruits and vegetables, the UADT cancer risk associated with lower educational levels was attenuated with only the levels of primary education or no education reaching statistical significance.^17^ In a pooled analysis of 23 964 HNC cases and 31 954 controls, Conway et al reported that lower educational levels were associated with an increased HNC risk. The elevated risk was largely attenuated by the adjustment for cigarette smoking and alcohol drinking, which explained two‐thirds of the elevated HNC risk.^6^ Although a large proportion of the increased HNC risk associated with lower SES was mediated through the major risk factors of HNC, the persisting significant association between lower SES and the elevated HNC risk after controlling for these mediators suggested that there are other unknown SES‐related factors that can affect HNC risk (Figure 2). Identifying these unknown SES‐related factors could help further decrease the incidence of HNC.

Although improving education might be important to reduce health disparity, our results further revealed that, under the same educational environment, there was a disparity in the association between alcohol use and HNC risk by educational level, whereas the association between the use of betel quids or cigarettes and HNC risk did not differ by educational level. This suggests that factors other than education could be important in determining health disparity in the association between these lifestyle factors and HNC. Although it is unclear what these other factors might be to explain our results, it is possible that they are due to actions taken by the Taiwan government to reduce the consumption of cigarettes and betel quids, particularly at the “upstream” level, including policies and social interventions. “Upstream” interventions tend to create fewer health inequalities compared to “downstream” interventions that focus on behavioral changes of individuals.^18^ The Tobacco Hazards Prevention Act (THPA) was enacted by the Taiwan government in 1997 with several subsequent amendments (https://law.moj.gov.tw/ENG/LawClass/LawAll.aspx?pcode=L0070021). The THPA includes tobacco health and welfare surcharges, regulations of retail sales, prohibition of advertising and product placement, a legal smoking age of 18 years, limitation of smoking locations, promotion of tobacco hazards, and penalties for violating smoking regulations. Since the implementation of the THPA, the prevalence of cigarette smoking in Taiwan has decreased from 29.2% in 1996 to 14.5% in 2017.^19^ Similarly, the Taiwan government has implemented various measures to reduce betel quid consumption, including: (i) internet and media campaigns to increase public awareness about the carcinogenicity of betel quid; (ii) betel quid cessation programs; (iii) collaboration with community leaders to build betel quid‐free living and working environments; and (iv) subsidies for aiding the conversion of betel quid farming to other crops.^19^ Consequently, the prevalence of betel quid use among Taiwanese men decreased from 17.2% in 2007 to 6.1% in 2017.^19^ Because of the public health efforts by the government and nongovernmental organizations, the majority of the Taiwanese recognize the harmful effects of cigarette and betel quid. According to the 2018 Taiwan Adult Smoking Behavior Survey, 82.2% of the survey subjects could name the diseases caused by cigarette smoking.^20^ The 2013 Taiwan National Health Interview Survey showed that 66.1% agreed that betel quid is carcinogenic.^21^ Since 2004, individuals who smoke cigarettes and/or chew betel quids (alcohol drinking is not one of the eligible criteria) have been invited to participate in the nationwide population‐based screening program for oral cancer, which accounts for more than 50% of the HNC occurring in Taiwan.^22^ Approximately 5% of the screening participants were diagnosed with oral premalignancy at first screening.^22^ The early detection and treatment of oral premalignancy through the nationwide population‐based screening program for cigarette and betel quid users might have also contributed partly to attenuate the health disparity in the association between the use of cigarettes or betel quids and HNC.

The only other study that examined the impact of SES on the association between lifestyle factors and HNC risk also reported a stronger positive association between alcohol and HNC among the lower educational group.^23^ This phenomenon is not unique to HNC. The term “alcohol harm paradox” refers to the phenomenon in which individuals with higher SES drink more alcohol but more alcohol‐related harm is disproportionally suffered by individuals with lower SES.^24^ In contrast to the declining use of betel quids and cigarettes, alcohol consumption has been increasing in Taiwan.^25^ In addition, alcohol control policies lag largely behind those for tobacco control in Taiwan, with insufficient regulation in alcohol taxation, advertising/product placement, product labeling, and the hours and locations of sales. Our result showing the health inequality in the association between alcohol use and HNC risk suggests the insufficient “upstream” interventions at the policy level for alcohol control in Taiwan.

Our results indicated that education had a stronger impact on the association between alcohol and HNC among carriers of the ALDH2*2 allele, who are genetically susceptible to the carcinogenic effect of alcohol. Taiwan has the highest prevalence (approximately 50%) of ALDH2*2 allele carriers in the world.^26^ This suggests that, compared to other countries, the same level of alcohol consumption will lead to higher alcohol‐related HNC burden in Taiwan. A study from Japan, which is another country with a high prevalence of ALDH2*2 allele carriers, reported that even light to moderate level of drinking was associated with an increased risk of cancer (OR for 10 drink‐years = 1.05; 95% CI, 1.04‐1.06 compared to never drinkers).^27^ In addition, those who drank 2 or fewer drinks per day had an increased cancer risk regardless of the drinking duration.^27^ Our results further indicated that, among those genetically susceptible to the carcinogenic effect of alcohol, lower educational levels further enhanced the risk of alcohol‐related HNC. These results reinforced the importance of devising public health strategies to decrease alcohol consumption across all SES levels in Taiwan, particularly strategies that target the low SES group, in order to reduce the health disparity in the association between alcohol use and HNC.

This study has some limitations. In a hospital‐based case‐control study, it is hard to know whether cases and controls came from the same source population. In addition, selection bias could be an issue to distort the effect estimate for the association between the exposure and the outcome. For our study, 97% of the study subjects were from Tainan City (88.0% of the cases and 88.3% of the controls) and Kaohsiung city (8.9% of the cases 8.4% of the controls). Compared to the 2013 National Health Interview Survey (NHIS) carried out in Taiwan,^28^ our controls had a similar educational level for men (70.2% of men in the NHIS completed at least a high school education vs 68.1% for our study) and a lower educational level for women (65.1% of women in the NHIS completed at least a high school education vs 47.5% for our study). As more than 90% of our study subjects were men, the bias toward the null for the association between education and HNC should have been minimal. Our male control subjects showed a similar percentage of current alcohol drinkers with that of men in the 2013 NHIS (2013 NHIS, 29.6% vs our study, 34.0%) whereas our female controls had a lower percentage of current alcohol drinkers than that of women in the 2013 NHIS (2013 NHIS, 9.7% vs our study, 3.3%).^28^ Again, as the majority of our study subjects were men, the bias for the association between alcohol and HNC should have been minimal. Our control subjects showed similar percentages of current betel quid chewers with those reported by the 2013 NHIS (2013 NHIS, 10.9% for men and 0.7% for women vs our study, 9.5% for men and 1.6% for women).^28^ For smoking, our control subjects had higher prevalence of ever smokers (those who had smoked at least 100 cigarettes in their lifetime) than that reported by the 2013 NHIS (2013 NHIS, 49.1% for men and 2.4% for women vs our study, 68.3 for men and 4.9% for women.^28^ This could have biased the association between cigarette smoking and HNC towards the null for both the high and low educational groups, partly explaining why we did not observe a significant influence of educational level on the association between cigarette smoking and HNC. Due to the case‐control study design, study participants were asked to recall lifestyle habits, including the use of alcohol, betel quids, and cigarettes. Inevitably, there could be some recall errors for reporting past exposures. Furthermore, cases were likely to ruminate more than controls about past exposures that might have resulted in their development of HNC, biasing the results away from the null. Another limitation is that we focused on education as the SES indicator. Although education is an important SES measure, it might not capture all aspects of SES. Previous studies using occupational class as an indicator of SES did not observe an association between the levels of SES and HNC.^7^, ^8^, ^9^ Educational level can determine an individual’s occupational class and health behaviors. Head and neck cancer is largely attributed to health behaviors, with alcohol and tobacco contributing to 72% of the cases, according to Hashibe et al.^29^ Because the association between education and health behaviors is more direct than that between occupation and health behaviors, education is perhaps better than occupation in capturing the health behavior aspect when investigating the association between SES and HNC. Another limitation is that, due to the differences in lifestyle behaviors, health policies, and socioeconomic structures, our results might not be generalizable to populations from other countries. Another limitation is the lack of lifetime exposure information for alcohol to provide a more complete picture regarding the impact of SES on the relationship between alcohol use and HNC. Finally, the study sample size might have a lower statistical power for detecting effect modification, particularly when the exposure variables were stratified into multiple categories.

This study has several strengths. This is one of the few studies to examine the influence of SES on the association between lifestyle behaviors and HNC risk. More importantly, this study was undertaken in a country with universal health care. The results suggested that that universal access to low‐cost quality health care might not completely mitigate the higher HNC risk associated with lower SES, especially those due to alcohol use, although further studies are required to directly confirm this relationship. Another strength of this study is the incorporation of the *ALDH2* SNP rs671 in our analysis, which indicated the possible role of education in influencing the association between alcohol and HNC among those genetically susceptible to the carcinogenic effect of alcohol. This supported that the difference in the association between alcohol and HNC by education level observed by the current study might not occur by chance, although more studies are needed to confirm this finding.

In conclusion, our study revealed a disparity in the association between alcohol and HNC by educational level. More studies are needed to confirm our findings and to determine the reasons for this disparity. One of the possible reasons to explain this disparity is the lack of sufficient alcohol control policies in Taiwan. Although the consequences of insufficient alcohol control policies need to be further evaluated, our results suggested that “upstream” interventions, including effective alcohol control policies, might be needed to reduce alcohol consumption and the health disparity in the association between alcohol and HNC.

## CONFLICT OF INTEREST

The authors have no conflict of interest.

## Supporting information

Table S1

Table S2
