# Supplementary material for: Investigating the health disparities in the association between lifestyle behaviors and the risk of head and neck cancer
Source: Cancer Sci. 2020 Jul 23;111(8):2974–86. doi: 10.1111/cas.14530 (PMC7419018; doi:10.1111/cas.14530)
Supplement: Supplementary file 1 — Table S1 [file CAS-111-2974-s001.pdf]

Table S1. The distribution of clinical diagnoses among controls

**Clinical diagnosis**	**N = 1,363**<br>**n**
Acute sinusitis	1
Aural polyp	1
Benign ethmoid tumor	2
Benign external auditory canal tumor	1
Benign hypopharyngeal lesions	13
Benign laryngeal lesions	41
Benign maxillary sinus lesions	11
Benign nasopharyngeal lesions	8
Benign neck tumor	47
Benign oral lesions	54
Benign oropharyngeal lesions	24
Benign parapharyngeal space tumor	2
Benign salivary gland tumor	192
Benign vocal cord lesions	170
Branchial cleft cyst	1
Chest abscess	1
Cholesteatoma	21
Chronic carditis	1
Chronic otitis media	99
Chronic rhinitis	35
Chronic sinusitis	326
Deep neck infection	1
Elongated styloid	1
Epiglottic cyst	17
Epistaxis	1
Esophageal stenosis	1
Ethmoid mucocele	3
External auditory canal osteoma	1
External auditory canal stenosis	4
Facial lipoma	1
Fungal sinusitis	6
Incomplete glottis closure	5
Laryngocele	1
Mastoiditis	2

Middle turbinate headache syndrome	2
Nasal polyp	5
Nasal septum deviation	1
Neck abscess	3
Neck lipoma	8
Neck lymphadenopathy	1
Neck lymphangioma	1
Obstructive sleep apnea	66
Oroantral fistula	1
Preauricular sinus	2
Ranula	3
Sialolithiasis	24
Thyroglossal duct cyst	21
Tonsillar hypertrophy	1
Tonsillitis	15
Torus palatinus	2
Tracheal granuloma	1
Tympanic membrane perforation	3
Vallecular cyst	2
Vocal cord atrophy	1
Vocal cord palsy	7
Vocal cord polyp	97
Wegener’s granulomatosis	1
