# Supplementary material for: Investigating the health disparities in the association between lifestyle behaviors and the risk of head and neck cancer
Source: Cancer Sci. 2020 Jul 23;111(8):2974–86. doi: 10.1111/cas.14530 (PMC7419018; doi:10.1111/cas.14530)
Supplement: Supplementary file 2 — Table S2 [file CAS-111-2974-s002.pdf]

Table S2. The associations between educational or income levels and oral hygiene status and intake of vegetables and fruits.

	Case			Control		
	Education					
	≦ Junior high	≧ High school/Technical school	P^a	≦ Junior high	≧ High school/Technical school	P^a
**Oral hygiene score[b]**						
0, 1 (Good)	96 (15.6)	151 (31.3)	<0.0001	110 (24.7)	518 (56.5)	<0.0001
2 (Moderate)	251 (40.7)	186 (38.6)		203 (45.5)	300 (32.8)	
3 (Poor)	270 (43.8)	145 (30.1)		133 (29.8)	98 (10.7)	
**Fresh vegetables**						
≦once /week	27 (4.4)	12 (2.5)	0.01	6 (1.3)	11 (1.2)	0.13
2-4 times/week	95 (15.4)	50 (10.3)		40 (9.0)	55 (6.0)	
Daily	496 (80.3)	422 (87.2)		401 (89.7)	850 (92.8)	
**Fresh fruits**						
≦once /week	274 (44.4)	189 (39.0)	0.03	117 (26.2)	188 (20.5)	0.06
2-4 times/week	166 (26.9)	120 (24.8)		111 (24.9)	241 (26.3)	
Daily	177 (28.7)	175 (36.2)		218 (48.9)	487 (53.2)	
	**Monthly income**					
	< NT$ 60000	≧ NT$ 60000	P^a	< NT$ 60000	≧ NT$ 60000	P^a
**Oral hygiene score[b]**						
0, 1 (Good)	95 (18.0)	74 (33.6)	<0.0001	181 (40.6)	315 (57.2)	<0.0001

2 (Moderate)	207 (39.2)	97 (44.1)		177 (39.7)	181 (32.8)	
3 (Poor)	226 (42.8)	49 (22.3)		88 (19.7)	55 (10.0)	
**Fresh vegetables**						
≦once /week	22 (4.2)	3 (1.4)	0.07	10 (2.2)	3 (0.5)	0.06
2-4 times/week	78 (14.8)	26 (11.7)		31 (7.0)	35 (6.4)	
Daily	428 (81.0)	193 (86.9)		405 (90.8)	513 (93.1)	
**Fresh fruits**						
≦once /week	238 (45.3)	67 (30.2)	<0.0001	130 (29.1)	96 (17.4)	<0.0001
2-4 times/week	148 (28.1)	56 (25.2)		126 (28.3)	139 (25.2)	
Daily	140 (26.6)	99 (44.6)		190 (42.6)	316 (57.4)	

Abbreviations: N = number; SE =standard error

a. P-value was calculated using chi-squared test
b. Oral hygiene score = tooth brushing + use of dental floss + regular dental visit, with tooth brushing: ≧2 times per day=0, <2 times per day=1; Use of dental floss: yes=0, no=1; and regular dental visit: yes=0, no=1.
